# Identification of bioactive peptides from a Brazilian kefir sample, and their anti-Alzheimer potential in *Drosophila melanogaster*

**DOI:** 10.1038/s41598-022-15297-1

**Published:** 2022-06-30

**Authors:** Serena Mares Malta, Letícia Leandro Batista, Heitor Cappato Guerra Silva, Rodrigo Rodrigues Franco, Matheus Henrique Silva, Tamiris Sabrina Rodrigues, Lucas Ian Veloso Correia, Mário Machado Martins, Gabriela Venturini, Foued Salmen Espindola, Murilo Vieira da Silva, Carlos Ueira-Vieira

**Affiliations:** 1 Institute of Biotechnology, Federal University of Uberlândia, Uberlândia, MG Brazil; 2 Laboratório de Genética e Cardiologia Molecular-LIM-13, Instituto do Coração (InCor), Hospital das Clinicas HCFMUSP, Faculdade de Medicina, Universidade de São Paulo, São Paulo, Brazil; 3 Pró-Reitoria de Pesquisa e Pós-Graduação, Universidade Federal de Uberlândia, Uberlândia, MG Brazil; 4 Laboratory of Genetics, Institute of Biotechnology, Federal University of Uberlândia, Acre Street, 2E building, room 230, Uberlândia, MG 38405-319 Brazil

**Keywords:** Biochemistry, Biotechnology, Drug discovery

## Abstract

Alzheimer’s disease (AD) is the most common form of dementia in the elderly, affecting cognitive, intellectual, and motor functions. Different hypotheses explain AD’s mechanism, such as the amyloidogenic hypothesis. Moreover, this disease is multifactorial, and several studies have shown that gut dysbiosis and oxidative stress influence its pathogenesis. Knowing that kefir is a probiotic used in therapies to restore dysbiosis and that the bioactive peptides present in it have antioxidant properties, we explored its biotechnological potential as a source of molecules capable of modulating the amyloidogenic pathway and reducing oxidative stress, contributing to the treatment of AD. For that, we used *Drosophila melanogaster* model for AD (AD-like flies). Identification of bioactive peptides in the kefir sample was made by proteomic and peptidomic analyses, followed by in vitro evaluation of antioxidant and acetylcholinesterase inhibition potential. Flies were treated and their motor performance, brain morphology, and oxidative stress evaluated. Finally, we performed molecular docking between the peptides found and the main pathology-related proteins in the flies. The results showed that the fraction with the higher peptide concentration was positive for the parameters evaluated. In conclusion, these results revealed these kefir peptide-rich fractions have therapeutic potential for AD.

## Introduction

Alzheimer’s disease (AD) is a multifactorial progressive pathology and the most common form of dementia^1^. It compromises distinct cognitive abilities, such as learning and memory, motor performance, and visual-spatial function^2^. In 2019, AD caused 1.5 million deaths, and by 2050 global cases are estimated to reach 175.9 million^3^. It is neuropathological characterized by lesions and atrophy in brain regions and the main hypothesis for its development is the aggregation of beta-amyloid peptides^4^. Those 42 amino acid peptides are generated through the amyloidogenic pathway, in which the amyloid precursor protein (APP) is cleaved by a β-secretase (BACE) followed by a γ-secretase^4–6^.

Recent studies indicate that AD’s pathology is directly correlated to dysbiosis in the gut-brain axis, inducing inflammatory and oxidative responses, while also influencing β-amyloid aggregation^7,8^. In this context, probiotics administration have demonstrated a positive effect in AD animal models and patients^9,10^. Among those, kefir is an especially promising probiotic. It is constituted by symbiotic bacteria and yeasts, which species and ratio can vary according to geographical origin and substrate—most commonly cow milk^11,12^. In our previous studies, kefir *in natura* and its metabolites decreased amyloid aggregation and improved the motor capacity of a *Drosophila melanogaster* model for AD (AD-like flies)^13^. Kefir’s bioactive peptides, however, are still to be explored against AD.

*D. melanogaster* has been extensively used to explore AD’s neurobiology and possible treatments^14,15^. It has favorable genetic characteristics and toolboxes, complex behavior, and a simplified nervous system and gut-brain axis—although conserved in relation to higher organisms. It is a unique model for exploring probiotic treatments to attenuate Alzheimer’s symptoms, especially targeting the amyloidogenic pathway^16–19^.

In order to provide a better understanding of the effect of Kefir’s bioactive peptides in AD’s pathology, we treated the AD-like flies with kefir and evaluated behavior and oxidative stress markers, as well molecular docking and in vitro approach.

## Results

### Kefir proteome and peptidome

**Figure 1:**
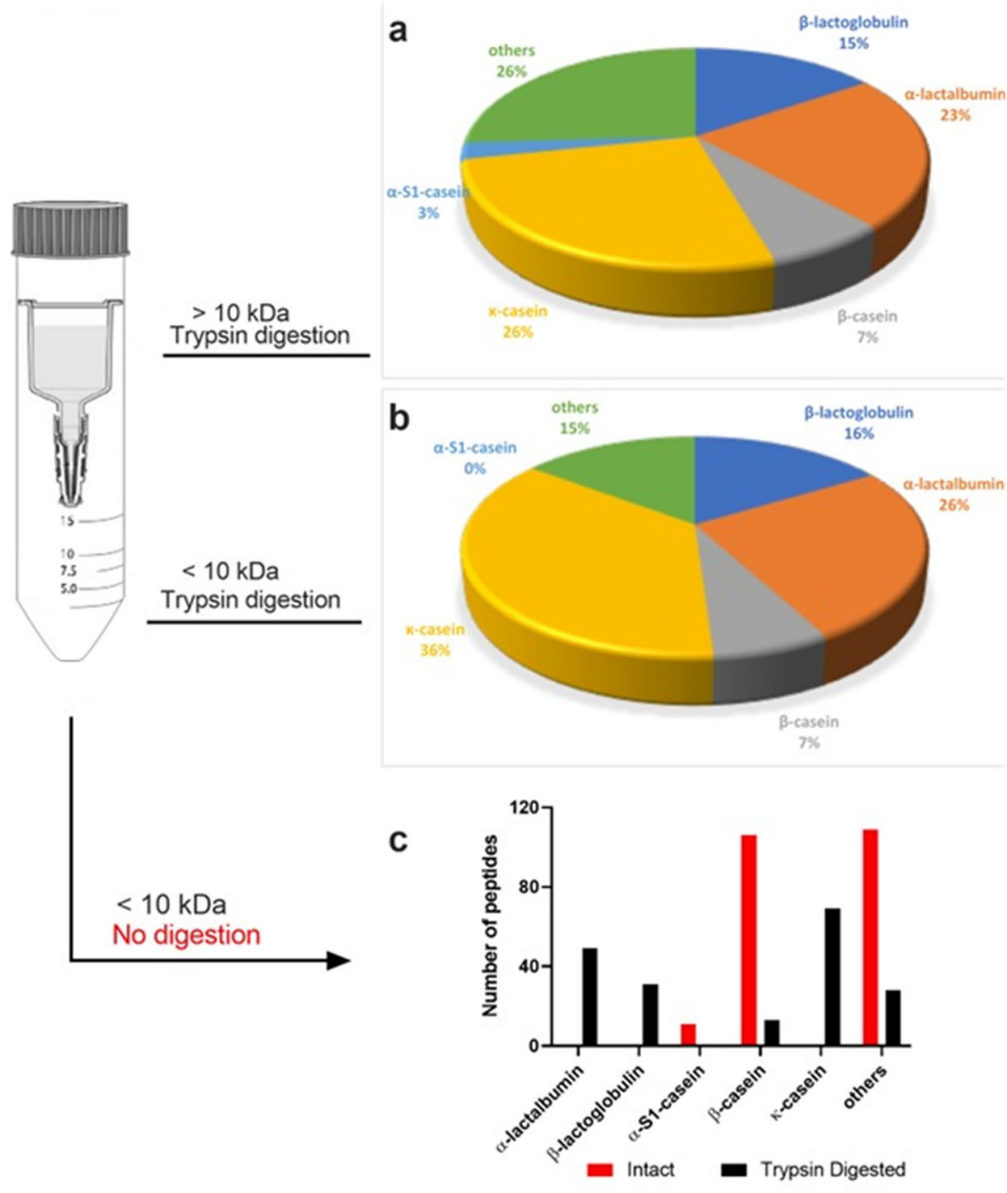
Proteome and peptidome of the kefir sample. (**a**) Proteomic analysis of the > 10 kDa fraction digested with trypsin and relationship of the proteins. (**b**) Proteomic analysis of the < 10 kDa fraction digested with trypsin and relationship of the proteins. (**c**) Peptidomic analysis of the < 10 kDa non-digested fraction and the quantity of peptides found that are derived from milk. Parts of the figure were drawn by using pictures from Servier Medical Art (https://smart.servier.com/).

Protein fractions (> and < 10 kDa) were analyzed by proteomic approaches. When the peptides were analyzed using the database “milk AND bovine”, a similar profile of peptides from milk proteins was found in both fractions, except for α-S1-casein, which was found only in the > 10 kDa fraction (Fig. 1a,b).

The peptidome analysis of < 10 kDa fraction was performed by mass spectrometry without trypsin digestion. Peptides derived from α-S1-casein, β-casein and only one from β-lactoglobulin were found in the main milk protein (Fig. 1c). Peptides were also found from the *Lactobacillus* and *Acetobacter* databases. However, in this work, we focused only on peptides encrypted in milk proteins.

### In silico prediction of bioactivities

The prediction of the physicochemical parameters and bioactivities of peptides from the “milk AND bovine” database and de novo analysis showed a large number of peptides with antioxidant, immunomodulatory, and ACE inhibitory activity, among others (Supplementary Table S1).

### In vitro analysis of kefir fraction

As a first step to analyze the diversity of kefir water-soluble fractions (WSF), the fractions were tested for their acetylcholinesterase inhibition and antioxidant capacity in vitro.

**Figure 2:**
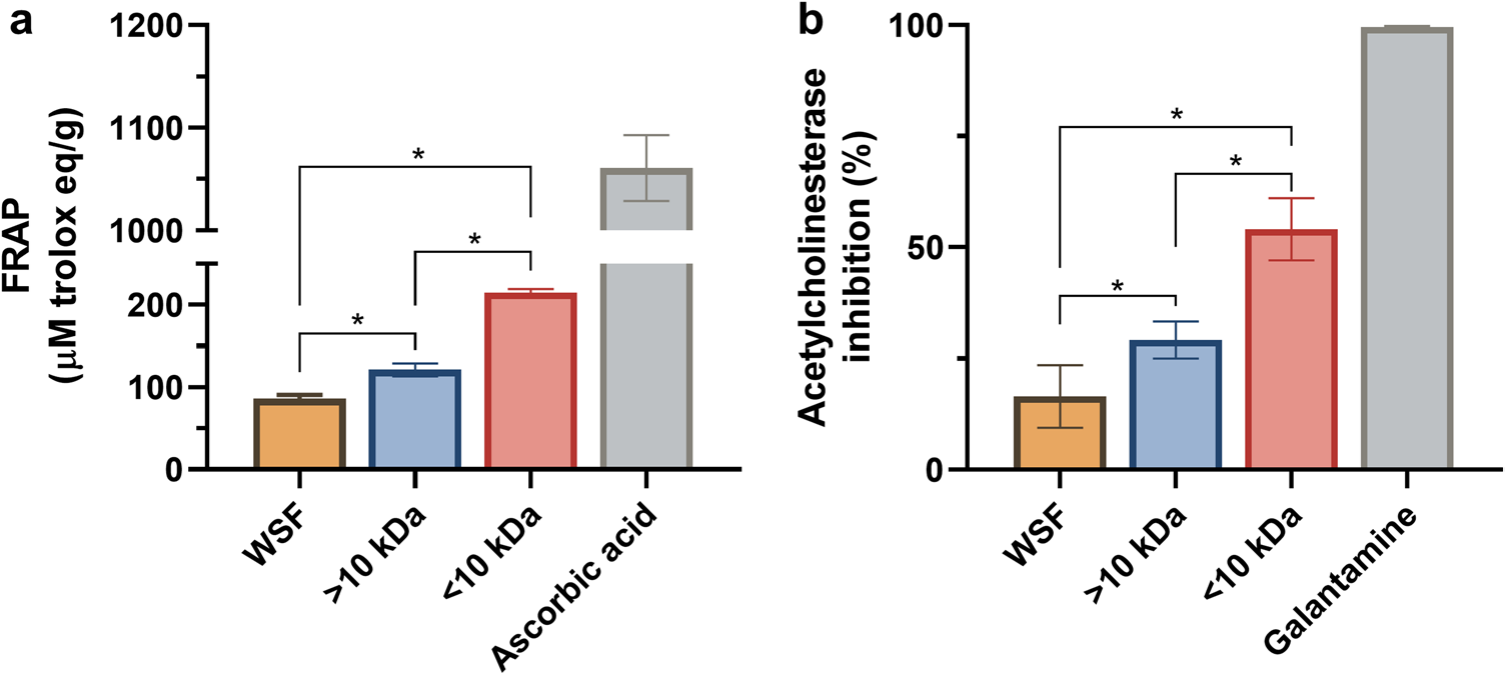
In vitro analysis of the effects of kefir fractions. (**a**) Total antioxidant capacity by the FRAP method of Fe^3+^ reduction analysis. The < 10 kDa fraction shows higher antioxidant activity compared to the others (*p < 0.5), but not as high as ascorbic acid used as the control. (**b**) Acetylcholinesterase enzyme inhibition capacity. Among the kefir fractions, the < 10 kDa fraction stands out as having the highest inhibition capacity (*indicates p < 0.5).

Ascorbic acid was used as a positive control for the FRAP test^20^; as a pure compound, it demonstrated the highest antioxidant capacity. In this in vitro antioxidant assay, all tested fractions, i.e. the WSF and peptides (> 10 kDa and < 10 kDa), demonstrated antioxidant capacity in terms of converting Fe^3+^ into Fe^2+^ (Fig. 2a). With improvements in the filtering and purification processes, the antioxidant capacity increased, with the < 10 kDa/peptidic fraction showing the best performance (p < 0.5) (Fig. 2a).

In the in vitro acetylcholinesterase inhibition assay, the fractions showed a similar pattern, with the peptidic fraction < 10 kDa showing the highest inhibitory activity (p < 0.5) (Fig. 2b). However, the WSF fraction did not inhibit acetylcholinesterase.

### *Drosophila melanogaster* AD model

After observing the promising activity of the fractions in preliminary in vitro studies, we used the fractions in a *D. melanogaster* model of Alzheimer’s disease (AD-like). This model is based on the expression of human BACE and APP, which mimics the amyloidogenic pathway in the fruit fly brain.

To ensure that the AD model is suitable for the evaluation of the fractions, we evaluated motor ability, performed β-amyloid quantification, and completed a histopathological analysis. For these assays, elav-Gal4/+ flies were used as the control.

**Figure 3:**
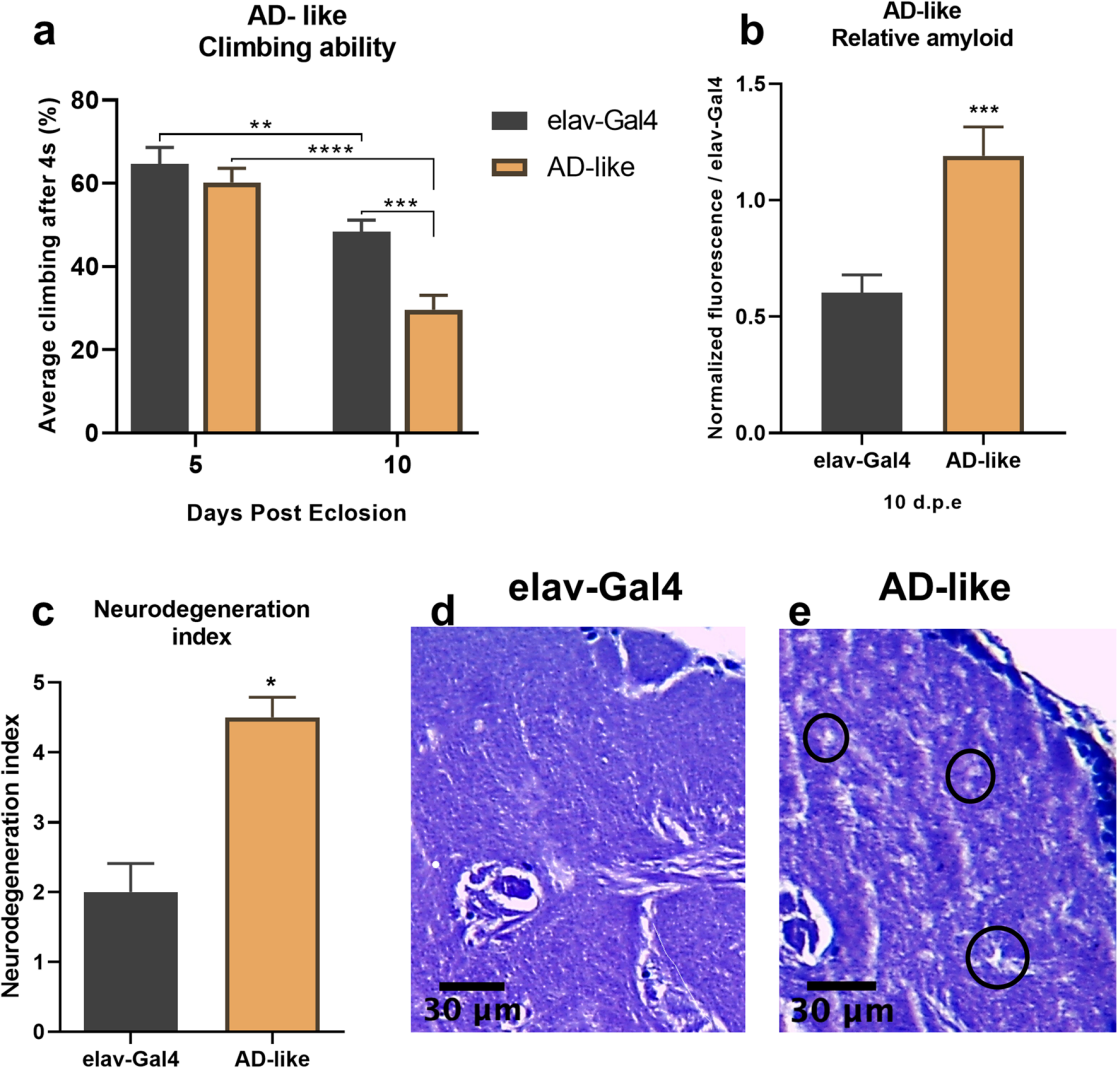
Validation of the Alzheimer’s disease model. (**a**) Climbing ability. AD-like flies show reduced motor ability compared to the control genotype at 10–13 days post eclosion (*n* = 90 in each genotype). (**b**) Quantification of amyloid by the Thioflavin T method. AD-like flies showed a higher amount of amyloid compared to the control at 10–13 days post eclosion (*n* = 30 for each genotype). (**c**) Neurodegeneration index of elav and AD-like flies based on the histopathological analysis, focusing on vacuolar lesions. Indices range from 0 to 5 with 0 indicating no lesions and 5 indicating a neurodegenerative phenotype. Data are presented as mean ± SEM, and significance values are represented as **p < 0.0001, *p < 0.001, **p < 0.01. Representative histopathological images of the elav (**d**) and AD-like (**e**) genotypes.

To evaluate a decline in motor reflex behavior, linked to neurodegeneration, a negative geotaxis assay was used. As expected, 10-to 13-day-old flies presented impaired motor performance when compared to the control genotype (p < 0.01) and to 5- to 8-day-old flies (p < 0.001) (Fig. 3a).

Using the amyloid binding capacity of Thioflavin T^21^, we quantified the relative amyloid levels in 10- to 13-day-old flies (*n* = 30). AD-like flies had a higher level of β-amyloid than control flies (p < 0.01, Fig. 3b).

Finally, the brains of 10-to 13-day-old flies underwent a histopathological analysis to evaluate the neurodegeneration index, ranging from 0 to 5, as observed in elav-Gal4 (Fig. 3d) and AD-like flies (Fig. 3e), in accordance with previous studies^13,22^. These results point to a greater degree of neurodegeneration in AD-like flies compared to control flies (p < 0.5, Fig. 3c).

### The peptide fraction from kefir can modulate the AD model

**Figure 4:**
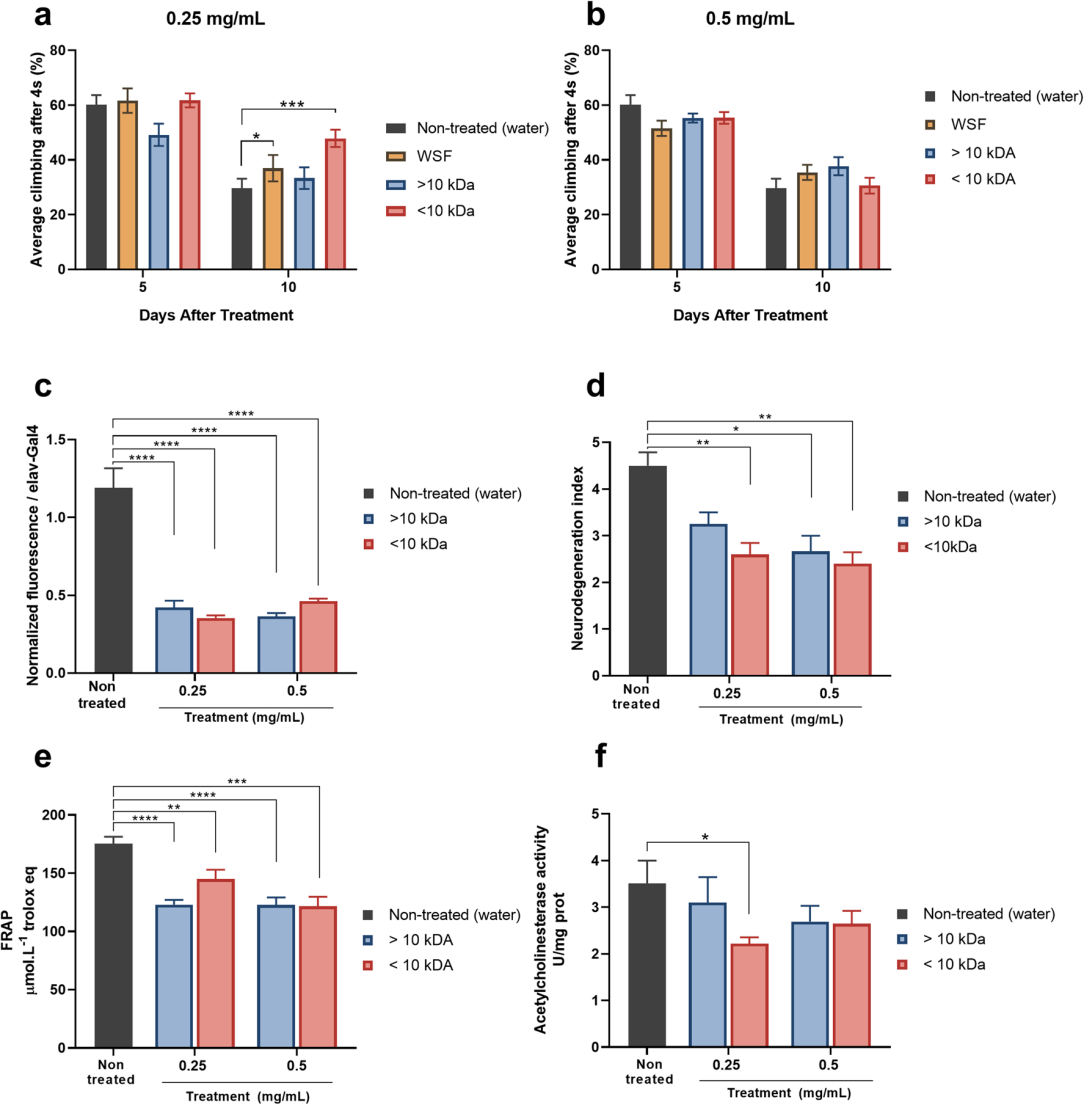
In vivo effects of kefir fractions. (**a**) The climbing ability of AD-like flies after 5 and 10 days of treatment with the fractions at a concentration 0.25 mg/mL, flies treated with WSF and the < 10 kDa fraction showed an improvement in motor performance (*n* = 90). (**b**) The climbing ability of AD-like flies after 5 and 10 days of treatment with the fractions at a concentration 0.5 mg/mL (*n* = 90). (**c**) Quantification of amyloid content by the Thioflavin T assay. Flies treated with all fractions at both concentrations showed a reduction in the amyloid content compared to the control (untreated) at 10 days of treatment (*n* = 30 for each treatment). (**d**) Index of neurodegeneration based on the histopathological analysis according to vacuolar lesions (*n* = 10 at 10 days of treatment). The results show a decrease in the index in all treated flies compared to the control. (**e**) Fe^3+^ reduction capacity by the FRAP method. Flies treated with the fractions show reduced antioxidant activity (*n* = 30 and 10 days of treatment). (**f**) Acetylcholinesterase activity. Only flies treated with the < 10 kDa fraction at 0.25 mg/mL demonstrated decreased acetylcholinesterase activity. Data are presented as mean + SEM. Statistically significant differences are indicated by *p < 0.5, **p < 0.1, ***p < 0.01 and ****p < 0.001.

Our previous work demonstrated a positive effect of kefir *in natura* and its compounds in this AD-like model^13^. In the RING assay, treated flies showed an improvement in climbing ability starting at 10 days after treatment (and therefore 10–13 days post eclosion) when treated with the WSF (p < 0.5) and < 10 kDa (p < 0.01) fractions at 0.25 mg/mL (Fig. 4a), compared to untreated flies. Treatment with fractions at 0.5 mg/mL did not show any improvements in climbing (Fig. 4b). In order to analyze the effect of the fractions in other parameters, follow up assays were done with flies at 10–13 days post eclosion, treated with < 10 kDa and < 10 kDa fractions at 0.25 and 0.5 mg/mL.

The t-test analysis showed that both fractions demonstrated a significant effect when administrated at 0.25 and 0.5 mg/mL. Flies treated with these samples showed a lower amyloid content (Fig. 4c) compared to control, neurodegeneration index (Fig. 4d) and antioxidant activity (Fig. 4e) when compared with untreated flies.

Exceptionally, in the anti-acetylcholinesterase assay (Fig. 4f), only flies treated with the < 10 kDa fraction (at 0.25 mg/mL) displayed a decrease in acetylcholinesterase activity when compared to control.

### Molecular docking

The < 10 kDa peptide fraction from kefir had antioxidant activity, improved climbing ability, decreased beta-amyloid levels and inhibited acetylcholinesterase activity. Molecular docking was performed to verify the peptides present in the fraction identified in the peptidomic assay in terms of BACE, amyloid fibrils and acetylcholinesterase.

**Figure 5:**
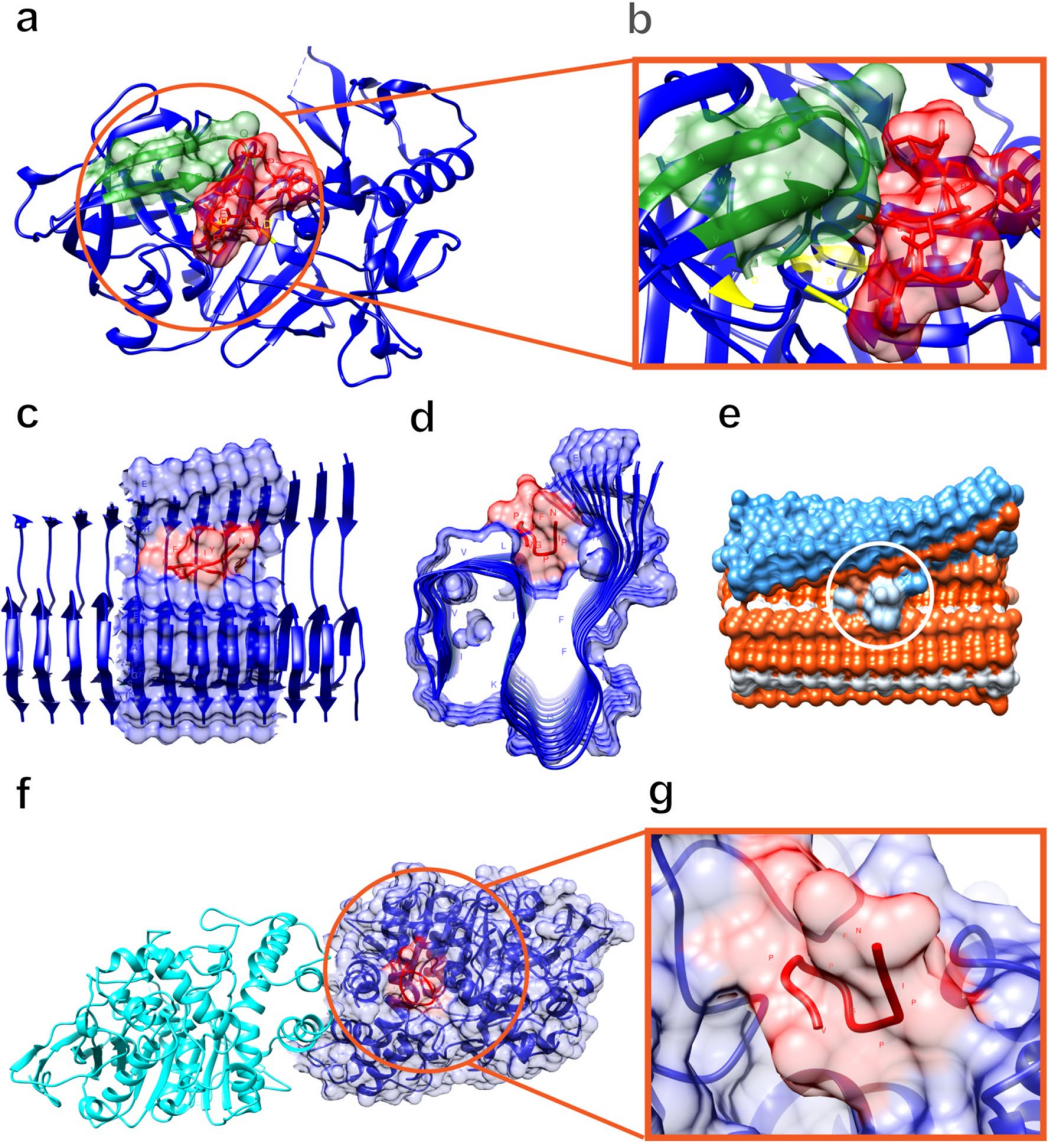
Molecular docking analysis of the VPPFLQPEV and VYPFPGPIPN peptides and their interaction with BACE1, Aβ and acetylcholinesterase. (**a**) Prediction of the interaction of the VPPFLQPEV peptide with the enzyme BACE1. (**b**) Zoomed in image of panel (**a**). (**c,d**) Prediction of the interaction of the VYPFPGPIPN peptide with β-amyloid plaques between the first and second β-strand regions. (**e**) Hydrophobic region of interaction of the VYPFPGPIPN peptide with β-amyloid plaque. (**f**) Prediction of the interaction of the VYPFPGPIPN peptide in the peripheral anionic site of the acetylcholinesterase enzyme. (**g**) Zoomed in image of panel (**f**).

Nine peptides were used for the prediction of 3D structure and docking. All peptides contributed atomic contact energy to the global energy (ACE) below − 6 in the docking prediction for BACE, Aβ(1–42) fibrils and human acetylcholinesterase (Table 1). The best model for docking (the peptide with the lowest ACE value for each protein) is shown in Fig. 5.

**Table 1:** Results of in silico docking of putative bioactivities from main milk proteins.

Source	Peptide Sequence	Peptide ranker	Protein	Docking (Kcal/mol)					
				BACE		β-amyloid		AChE	
				Global energy	ACE	Global energy	ACE	Global energy	ACE
DataBase	YPFVPGLP	0.86	β-casein	− 13.32	− 6.17	− 61.36	− 19.58	− 33.49	− 9.77
	VYPFPGPI	0.84	β-casein	No predicted					
	VAPFPEVFG	0.77	α-S1-casein	− 25.70	− 7.90	− 92.21	− 16.35	− 75.29	− 12.79
	EMPFPK	0.76	α-S1-casein	No predicted					
	LVYPFPGPI	0.74	β-casein	− 41.44	− 10.85	− 82.06	− 20.24	− 73.62	− 14.66
	VYPFPGPIPN	0.72	β-casein	− 37.34	− 11.95	− 78.37	− 24.27	− 70.68	− 14.72
	SLPQNIPPLTQTPVVVPPFL	0.68	β-casein	− 35.08	− 7.61	− 104.61	− 20.23	− 20.66	7.45
De novo	HQPHQPLPPT	0.62	β-casein	− 23.83	− 4.29	− 50.56	− 8.54	− 57.54	− 9.95
	VPPFLQPEV	0.53	β-casein	− 44.55	− 13.33	− 96.41	− 19.06	− 57.70	− 8.75

The peptide VPPFLQPEV was predicted as the best ligand for BACE. This peptide binds to an important flap that controls enzyme activity by partially covering the substrate-binding cleft localized between the N- and C-terminus lobes (Fig. 5a,b).

The best predicted ligand for Aβ(1–42) fibrils was the peptide VYPFPGPIPN. It could bind between the first and second β-strand regions (Fig. 5c,d). This is a hydrophobic region, and the binding of the peptide in this region may make it more hydrophobic (Fig. 5e).

The peptide VYPFPGPIPN was also predicted as the best ligand for human AChE. This enzyme has two binding sites: one is a peripheral anionic site (PAS) located at the entrance of the active gorge and the other is a catalytically active site (CAS) located at the base of the active site gorge. The peptide bound to PAS (Fig. 5f,g).

## Discussion

Kefir is a broadly used probiotic, characterized as a symbiotic agglomerate of bacteria and yeasts. It is popularly used as a complementary treatment in many diseases^23–26^. Throughout the milk fermentation process, kefir microorganisms present a unique protein metabolic process, resulting in bioactive molecules^27^ among these peptides. The composition of kefir grains varies according to geographic location and fermentation parameters^11,28,29^ and is thought to contribute to variations in its metabolites and peptides. Even though there is variability, some core bacteria and yeasts remain consistent across samples, which allows for the comparison of different studies^12^. Our group has already characterized the effects of raw kefir and its organic fractions—as well as its metabolome—in improving AD-like flies^13^. This present work is a complementary effort, investigating the effects of kefir peptides in AD-like flies, along with a proteomic and peptidomic characterization. Additionally, this is the first study, to our knowledge, to characterize both peptides and metabolites from a single kefir grain sample.

In the present work, we focused on the peptidome of the kefir and in the intact bioactive peptides. The fermentation-generated peptides were identified through a proteomic analysis. Similar results have been described by other research groups^30,31^, which supports the compositional consistency of kefir despite some variations across samples.

As we used cell-free protein to produce the proteomic profile, the peptidases found using databases from *Lactobacillus* and *Acetobacter* could be from cells disrupted (intracellular) by fermentation or by a novel secretory protein degradation system^32^. Further assays are needed to resolve this question. In previous work, only these two bacteria genera were found in our kefir sample^13^.

Our in silico analysis indicates that the major part of the peptides generated from our kefir sample during fermentation display several activities, the characteristics of which have been described in other milk-derived peptides^33,34^. Here, we focused on putative acetylcholinesterase inhibition and antioxidant properties as well BACE inhibition and putative amyloid fibril binding.

These characteristics are especially interesting for AD. Since AD is a multifactorial disease^35^, researchers have turned their attention to developing multi-target drugs to inhibit the myriad factors involved in AD, including protein misfolding and associated Aβ aggregation, t aggregation, metal dyshomeostasis, oxidative stress and a decrease in AChE levels. Even though the proposed model is based on the amyloidogenic hypothesis, we should expect alterations in other metabolic processes. Previous studies have shown a relationship between amyloid aggregation and both oxidative stress^36^ and acetylcholinesterase activity^37^ during AD progression.

Oxidative stress is related to the neuropathological manifestations of AD and implies an increased level of reactive oxygen species (ROS)^38,39^ and the abnormal homeostasis of bioactive metals^40^. Otherwise, acetylcholinesterase catalyzes acetylcholine conversion, which is related to the cholinergic cascade and cholinergic neuron loss in AD pathology^41^.

In an effort to target those processes, antioxidant compounds (e.g. resveratrol) have shown a role in AD prevention^42^ or as a supportive treatment^43^. Furthermore, many AD drugs inhibit acetylcholinesterase activity, aiming to increase acetylcholine levels in the brain^44^. However, these drugs have a limited effect and generate collateral effects^45^.

Therefore, with a positive prediction from the in silico investigation, we screened the effect of the kefir fractions in vitro. Both antioxidant and anti-acetylcholinesterase properties were confirmed using the FRAP and acetylcholinesterase inhibition assays. To the best of our knowledge, this is the first work to report the anti-acetylcholinesterase activity of a kefir-produced molecule.

In order to verify the in vivo effects of peptides from kefir, we used the *D. melanogaster* AD-like model. For that, we first provided a confirmation of the model, in which human BACE and APP are overexpressed in fly neurons by the pan-neural driver elav-Gal4.

In the functional assays, the flies displayed a motor deficit starting at 10–13 d.p.e, which was correlated with a increase in amyloid relative level, demonstrated by amyloidogenic quantification, and severe neurodegeneration based on the histopathological analysis. This has been previously described for a similar model^13,46,47^ and our previous data with the model used here^48^, which indicates the suitability of the model.

With a validated model in hand, we treated AD-like flies with kefir fractions. Flies treated with the WSF and < 10 kDa fractions (both at 0.25 mg/mL) displayed better climbing ability in relation to untreated flies. However, the < 10 kDa fraction had an apparently stronger influence on this process, probably due to its higher peptide concentration due to the serial filtration process. However, although the > 10 kDa fraction improved the FRAP results, the neurodegeneration index and relative Aβ peptides, it was not able to inhibit acetylcholinesterase. Dose dependency was not observed here, which is possible since both fractions were constituted of a mix of peptides and polypeptides. The higher tested concentration (0.5 mg/mL) likely contained bioactive peptides against other targets can act in another sites. Again, only the lower concentration of the > 10 kDa fraction was able to generate a significant decrease in AChE activity.

To investigate this effect, first the peptides present in the < 10 kDa fraction were identified by LC–MS/MS. Then, the 3D peptides were predicted and finally molecular dockings was performed to identify potentially bioactive peptides with AChE, BACE1, and Aβ peptides as the targets. AChE has two binding sites: one is a PAS located at the entrance of the active site gorge, and the other is a CAS located at the base of the active site gorge^49^; ligands of PAS or CAS can inactivate this enzyme^50^. The binding of the VAPFPEVFG peptide at the PAS could be enough to promote the inhibition of acetylcholinesterase. Our docking data are only a tentative explanation for the characteristics and effects of kefir peptides. The real effects observed in vivo in the AD-like model were likely promoted by a complex mix of peptides.

The BACE1 molecule is formed of three portions: an extracellular N-terminal domain, a transmembrane domain and a C-terminal cytosolic domain. Its cleavage site is between the N- and C-terminal regions, characterized by an aspartate catalytic dyad (Asp32 and Asp228, highlighted in yellow in Fig. 5a,b)^51^. Near this region, there is a flexible flap (highlighted in green in Fig. 5a,b), perpendicular to the active site. This flexible flap can exist in either an open or closed conformation, and in that way help or hinder the access of a molecule to the enzyme active site^52,53^. Between the flap and the active site, there is also a space in the BACE1 structure that can be reached by a molecule so that it can access the catalytic dyad^54^.

By analyzing the 3D structure of BACE1 and the peptide VPPFLQPEV (Table 1 docking of smaller global energy value, Fig. 5a,b), it was possible to verify that it did not interact with the BACE1 active site. Despite this, the peptide interacted with ALCA, and was able to alter its conformation, strategically located between the large N- and C-terminal portions of BACE1. This peptide position could block the access of other molecules (substrates) to the enzyme active site, thereby inhibiting its action. As a consequence, BACE1 could lose its capacity to cleave APP, consequently reducing Aβ peptide production and amyloid plaque accumulation. In this way, it could be possible to decrease the progression of neurodegeneration through the amyloidogenic pathway.

The amyloidogenic pathway results in the production of Aβ peptides of distinct sizes, depending on the γ-secretase cleavage region. Amongst those, the 42 amino acid peptide (Aβ42) is the most neurotoxic^55,56^. Based on that, we also evaluated molecular docking between the bioactive peptide VYPFPGPIPN and amyloid plaques generated by the Aβ42 peptide.

This peptide has a unique conformation, composed of three β-sheets that cover the residues 12–18 (β1), 24–33 (β2) and 36–40 (β3)^57^. This tertiary fold is responsible for the toxicity and aggregation of this peptide, which results in β-amyloid plaque formation in AD. Based on the molecular docking analysis between the peptide VYPFPGPIPN and amyloid plaques (Fig. 5c,e), we observed an interaction between those molecules, and a consequent conformational change in the amyloid plaque, which was highlighted by the opening of its tertiary fold (Fig. 5e). This alteration in β-amyloid plaque conformation could reduce its toxicity, thus neutralizing the negative effects of its aggregation and therefore attenuating the effects of AD.

The present study extends the existing literature by providing evidence that peptides derived from cow milk kefir can modulate the AD phenotype in AD-like flies by decreasing the relative β-amyloid level in the brain. Consequently, this intervention decreases neuronal tissue damage, improves motor ability and decreases acetylcholinesterase activity. In summary, our study was able to identify bioactive peptides present in kefir (< 10 kDa fraction) and to predict their potential use as an alternative adjuvant treatment for AD or as a drug prototype.

## Methods

### Samples

Kefir grains were obtained as a donation from the local population in Uberlândia, Brazil, inoculated (4% w/v) in UHT (ultra-high temperature) whole cow milk for fermentation process and left for 24 h at room temperature in a glass container. A genetic fingerprint was previously published by our group^13^. The fermented product was collected by removing the grains by filtration, followed by centrifugation at 4 °C, 4900×*g* for 10 min. The resulting supernatant went through a series of filtering processes, where we obtained the three fractions that were further tested.

The pellet was discarded, and the supernatant was re-centrifuged, generating the kefir water-soluble fraction (WSF). This was then filtered using a vacuum pump and passed through a 0.22 µm membrane. Finally, this filtrate was passed through a 10 kDa Amicon column, separating peptides by size: larger than 10 kDa (referred to as > 10 kDa) and smaller than 10 kDa (< 10 kDa).

### Proteopeptidomics

In preparation for shotgun proteomics, the samples were reduced with 100 mM dithiothreitol (DTT) and alkylated with 0.5 M iodoacetamide. Samples were then digested with trypsin (0.01 μg/μL), except for the < 10 kDa peptide fraction, which was obtained from Amicon filtering. A desalination step was performed with a ZipTips C18 column (Millipore, Billerica, MA, United States).

Shotgun proteomics and peptidomics were performed by liquid chromatography–electrospray ionization–quadrupole–time of flight-mass Spectrometry (LC–ESI–Q–TOF–MS) using the 6520B instrument from Agilent. An AdvanceBio Peptide Mapping column (Agilent) was used (2.1 mm internal diameter, 10 cm long, 2.7 µm particles). In the mobile phase, water (A) and acetonitrile (B) were acidified with formic acid (0.1% v/v), using the gradient: 2% B (0 min), 2% B (10 min), 15% B (40 min), 50% B (150 min), 70% B (200 min), 98% B (220 min), 98% B (300 min), 100% B (301 min) and 100% B (400 min) at 400 µL/min. For ionization, a nebulizer pressure of 45 psi was used, with drying gas at 8 L/min (325 °C). 4 kV energy was applied to the capillary.

Spectrum Mill software (Agilent) was used for data analysis, using the following Uniprot databases: (1) “milk AND bovine” (69,760 results in February 2021); (2) “*Lactobacillus*” (1,250,616 results in February 2021) and (3) “*Acetobacter*” (185,991 results in February 2021). Carbamidomethylation was set as fixed with and. Maximum missed cleavages were selected in two for trypsin. The precursor mass error and the fragments at 20 ppm, product mass tolerance at 50 ppm, and maximum ambiguous precursor charge at 3.

For de novo analysis, the Sherenga module of the Spectrum Mill software (Agilent) was used.

### In silico physicochemical parameters, toxicity, and bioactivity prediction of obtained peptides

ToxinPred (https://webs.iiitd.edu.in/raghava/toxinpred/algo.php) was used to calculate the physicochemical characteristics of each characterized peptide and the expected toxicity level, while the Peptide Property Calculator from Innovagen (http://www.innovagen.com/proteomics-tools) was used to analyze solubility. For the bioactivity prediction, PeptideRanker (http://distilldeep.ucd.ie/PeptideRanker/) was used. Finally, the Milk Bioactive Peptide Database (http://mbpdb.nws.oregonstate.edu/) was used to determine the origin of peptides from milk protein and their putative biological functions. The peptides with a rank above 0.5 were considered as having potential bioactivity.

### In vitro fraction evaluations

#### Total antioxidant activity

Total antioxidant capacity was evaluated by FRAP (ferric reducing antioxidant power) assay^20^. This method consists of evaluating the capacity if a compound reduction Fe^3+^ to Fe^2+^ Kefir fractions (WSF and both Amicon samples) were solubilized in distilled water at 500 µg/mL. Ascorbic acid was used at the same concentration as the positive control and sodium acetate buffer was used as the blank.

For the assay, 250 µL of FRAP reagent (10× sodium acetate 0.3 M, TPTZ (2,3,5-triphenyltetrazolium chloride) 10 mM, 1 volume of ferric chloride 20 mM) were mixed with 10 µL of each sample and 25 µL of MilliQ Water. The reaction progressed for 6 min at 37 °C. The respective absorbances were measured at 593 nm on a spectrophotometer. The antioxidant capacity of each sample was determined by the construction of an analytical curve built with Trolox (6-hydroxy-2,5,7,8-tetramethylchroman-2-carboxylic acid).

#### Acetylcholinesterase inhibition assay

The acetylcholinesterase inhibition assay was based on the one described by Rhee^58^. In this assay, three Tris–HCl buffers were used: (I) standard Tris–HCl buffer (pH 8); (II) Tris–HCl with bovine serum albumin 1% w/v; and III) Tris–HCl with NaCl 0.6% w/v and MgCl_2_ 0.4% w/v. The enzyme was diluted (0.2 U/mL) in buffer I.

The solutions were prepared in the following way: the enzyme was diluted to 0.2 U/mL in buffer I, DTNB 0.1% was diluted in buffer III, acetylcholine iodide solution (substrate) 0.4% v/v was diluted in Milli-Q water, and the inhibitor Galantamine (positive control) diluted in Milli-Q water.

For the assay, 25 µL of each sample were added to 125 µL of DTNB solution, 50 µL of buffer II, 25 µL of acetylcholine iodide solution and 25 µL of acetylcholinesterase solution. Using a spectrophotometer, the absorbance was measured at 405 nm for 20 min at 30 °C. Acetylcholinesterase inhibition was calculated using the following equation:

$$Inhibition\left( \% \right) = \left[ {\left( {100 - \left( {Sample\;abs. - Blank\;abs.} \right)/\left( {Blank\;abs.} \right)} \right]} \right. \times 100.$$

### In vivo experiments

#### *Drosophila melanogaster* genetics

Fly stocks were obtained from the Bloomington Stock Center: *w*^1118^ (stock# 3605), UAS-BACE1, UAS-APP (#33797) and elav-GAL4 (#5146). Flies were kept in an incubator at 25 °C in a 12 h:12 h light:dark cycle. Unless otherwise stated, flies were reared on cornmeal medium (soy powder 0.01%, glucose 7.2%, agar 0.6%, cornmeal 0. 073%, yeast 0.018%, nipagin 0.06% and acid solution 0.05% w/v).

AD-like flies expressing human BACE and APP pan-neuronally were obtained by crossing elav-GAL4 and UAS-BACE, UAS-APP flies. Resulting pupae were selected according to phenotype—without any genetic marker, as tubby—ensuring that the resulting fly genotype is elav-Gal4/+; UAS-BACE, UAS-APP/+ (AD-like model).

#### Treatments

Fly treatment was administered via the food and began at 0–3 days post eclosion. All kefir samples were solubilized at 0.5 and 0.25 mg/mL. For this, 5 mL of sample was mixed into 1 g of enriched mashed potato medium (75% instant mashed potato, 15% yeast extract, 9.3% glucose, and 0.07% nipagin). The treatment medium was changed every other day to ensure fresh exposure to the samples. Control flies were fed with enriched potato powder medium solubilized with distilled water only. For all experiments, only male flies were used.

#### Rapid iterative negative geotaxis assay (RING)

The rapid iterative negative geotaxis assay (RING) was adapted from Gargano et al.^59^ and used to evaluate the effect of different types of treatment on fly locomotor ability. To ensure an AD-like phenotype, these flies were compared to control flies (elav-Gal4/+). Groups of 30 AD-like flies (in triplicate) for each treatment (WSF, 0.22 µm, and both Amicon conditions) were transferred to clean vials (9.5 cm × 2.5 cm) and placed in a custom vial holder. Flies had their behavior assessed 5 and 10 days after treatment. Before testing, flies were exposed to light and kept in a silent environment for 20 min, to acclimate. For the assay, the holder was hit three times on the bench, and the flies were given 4 s to climb 5 cm. This was repeated five times. The procedure was recorded, and the video was analyzed using QuickTime Player 7.7.9 software. The average climbing percentage was calculated as the percentage of flies of each group that reached the 5 cm mark 120 frames (4 s) after the holder touched the bench.

#### Total antioxidant activity in vivo

To evaluate the total antioxidant activity for each treatment, the modified FRAP method was used. Ten fly heads (in triplicate) after 10 days of treatment were homogenized with PBS and centrifuged (2 min, 1000×*g* at 4 °C). For the assay, 10 µL of each supernatant was mixed with 50 µL of FRAP reagent 1:1 in distilled water. Antioxidant activity was evaluated as previously described for the in vitro analysis.

#### Acetylcholinesterase activity assay

For the assay, homogenized fly head solutions were made as described above. Then, 25 µL of the solution was added to 50 µL of Tris–HCl buffer (bovine serum albumin 1% w/v), 125 µL of DNTB, 25 µL of acetylcholine iodine solution and 25 µL of acetylcholinesterase (0.2 U/mL) (based on Ellman’s protocol)^60^. Sample absorbance was measured at 405 nm at 30 °C for 20 min at 30 s intervals.

#### Amyloid quantification

The amyloid content was assessed using Thioflavin T (ThT), a benzothiazole dye that exhibits enhanced fluorescence upon binding to amyloid fibrils. Fly heads from elav-Gal4/+ (control) and AD-like flies (a pool of 10 heads each, in triplicate) were collected, frozen in liquid nitrogen, and kept at − 80 °C until the next step. Fly heads were then kept on ice and homogenized in PBS 1×, then centrifuged (2 min, 10,000×*g* at 4 °C). The supernatants were collected and used for amyloid quantification and Bradford protein levels in technical triplicates. In a protocol modified from Westfall et al.^61^ supernatants were incubated with the ThT working solution (20 µM) for 20 min under agitation. Fluorescence was measured at 450 nm excitation/482 nm emission and normalized to ThT only samples. Total fluorescence was corrected to the total protein content of each sample and to elav-Gal4/+ (control flies) fluorescence levels. This additional step was needed as in vivo samples could present autofluorescence.

#### Histopathological analysis

Ten flies were used from each treatment group, using the optimal concentration obtained in previous experiments. All flies were analyzed at 10–13 days post eclosion (d.p.e.) and elav-Gal4/+ was used as the control. Fly heads were transferred to 4% formaldehyde in sodium phosphate buffer 0.1 M pH 7.2 for 16 h at 4 °C. The samples were then dehydrated in a graded ethanol series (70, 80, 90, and 95%) and transferred to methanol for 16 h at 4 °C. Next, they were embedded in HistoResin (Leica) and 3 µm thick slices were stained with hematoxylin and eosin, analyzed and photographed with a light photomicroscope. Neuropile images were used to calculate the neurodegenerative index as normal to low, moderate or severe, according to vacuolar lesions^13,22^.

### Molecular docking

Putative bioactive components were used for docking analysis. The 3D peptide structures were created using PEP-FOLD 2.0. The Protein Data Bank (PDB) files for BACE (3TPJ), acetylcholinesterase (AChE-3LII) and 42-Residue Beta-Amyloid Fibril (2MXU), were retrieved from PDB. The docking between the peptides and these enzymes was performed using PathDocking. The best model was chosen based on both global energy and atomic contact energy contribution to the global binding energy.

### Statistical analysis

Data analysis was performed using GraphPad Prism 8. A priori, we evaluated the normal distribution of the data by the D’Agostino and Pearson test. Groups were compared through a t-test with an established significance level of p < 0.05.

## Supplementary Information

Supplementary Table S1.

## Data Availability

The datasets generated during and/or analyzed during the current study are available from the corresponding author on reasonable request. The raw data from mass spectrometry analyses were deposited in PRIDE access number: PXD034190 (fraction < 10 kDa digested with trypsin), PXD034189 (fraction > 10 kDa digested with trypsin) and PXD034148 (intact fraction < 10 kDa).
